# Study protocol and pilot study results for a clinical intervention trial of PKU carriers and non-carriers: the Phe for Me trial

**DOI:** 10.1186/s13023-025-04131-2

**Published:** 2026-01-19

**Authors:** Sophia M. Khan, Madison L. Fennell, Mazyar Fallah, Heather Jordan, Zachary Kroezen, Philip Britz-McKibbin, Philip J. Millar, Robyn R. Heister, Marie-Claude Vohl, Justine R. Keathley

**Affiliations:** 1 College of Biological Sciences, Department of Human Health Sciences, University of Guelph, Guelph, ON Canada; 2 Human Cardiovascular Physiology Laboratory, Department of Human Health Sciences, University of Guelph, Guelph, Canada; 3 Department of Chemistry and Chemical Biology, McMaster University, Hamilton, ON Canada; 4 Doctronic, San Francisco, California USA; 5 Centre Nutrition, santé et société, Institut sur la nutrition et les aliments fonctionnels, Université Laval, Québec, QC Canada

**Keywords:** Phenylketonuria, PKU, Genetics, Rare disease, Nutrition, Phenylalanine, Metabolism, Clinical trial

## Abstract

**Background:**

Phenylketonuria (PKU) is an autosomal recessive genetic condition caused by a PAH gene mutation that results in impaired function of the phenylalanine hydroxylase (PAH) pathway. Thus, L-phenylalanine (Phe) cannot be effectively hydroxylated into L-tyrosine (Tyr), so without treatment, Phe levels accumulate while Tyr levels remain low. While PKU is relatively well understood, there is currently sparse literature regarding the putative health and metabolic impacts among PKU carriers, who have been historically described as “unaffected.”

**Methods:**

This article provides a detailed overview of the methods for a single-arm clinical trial of PKU carriers and non-carriers, as well as results from a pilot feasibility study. This trial aims to determine whether PKU carriers, following an oral Phe challenge, exhibit an intermediate phenotype of PKU that may perturb metabolic, physiological and/or cognitive function. In this study, PKU carriers and non-carriers were recruited and following baseline measurements of chronic mental health, cognition, blood pressure, acute mood, and minimally invasive biospecimen collection, participants ingested a 100 mg/kg dose of Phe. Two hours post-Phe consumption, baseline measurements were repeated (except chronic mental health measures) to determine potential treatment response differences between carriers and non-carriers of PKU. The pilot study aimed to evaluate feasibility of the methods, and the small sample size limited statistical power; as such, no statistical analyses were conducted.

**Results:**

The pilot study demonstrated protocol feasibility and several intriguing but highly preliminary data trends. Among genetic carriers of PKU, we observed higher chronic impulsivity scores, and following the oral Phe challenge, greater increases in Phe/Tyr ratio in dried blood spot extracts, both systolic and diastolic blood pressure, as well as worsening acute mood scores compared to non-carriers.

**Conclusions:**

Overall, this research aims to enhance our understanding of the impact of nutrition on carriers of autosomal recessive inborn errors of metabolism who are presumed to be unaffected, starting with a focus on PKU. Pilot study results demonstrated feasibility of the protocol and some interesting preliminary data trends, however further studies that are adequately powered with larger sample sizes are needed to corroborate these findings.

**Clinical trail no:**

Phe For Me? ClinicalTrials.gov. NCT06119048. Registered 31 October 2023 - https://clinicaltrials.gov/study/NCT06119048

**Supplementary Information:**

The online version contains supplementary material available at 10.1186/s13023-025-04131-2.

## Background

Phenylketonuria (PKU) is a rare genetic disorder characterized by an inborn error in the metabolism of the amino acid *L*-phenylalanine (Phe) [1]. Individuals with PKU exhibit reduced enzymatic activity of phenylalanine hydroxylase (PAH), which converts Phe into *L*-tyrosine (Tyr) and other downstream metabolites [1]. PKU is an autosomal recessive condition, meaning that both parents must carry a mutated *PAH* gene, which is inherited by the offspring for the condition to manifest [2]. In Ontario, Canada, PKU affects approximately 1 in every 12,000 individuals, with similar rates across Canada, however disease prevalence varies by country and ethnicity [3, 4]. In contrast, carriers of PKU are more common with a prevalence of approximately 1 in 50 individuals (2%) [5]. PKU carriers are heterozygous for the mutated *PAH* gene, and emerging evidence suggests that these individuals may exhibit a milder phenotype of PKU with potential sub-clinical effects, and may not be considered as “unaffected carriers,” as is often stated in the literature. For example, early studies have indicated that among PKU carriers, PAH enzymatic functioning assessed via liver biopsies is reduced to about 7–10% of normal PAH enzymatic functioning [6]. Other reports have demonstrated metabolic perturbations in the PAH pathway (Phe to Tyr conversion) in PKU carriers vs. non-carriers following Phe consumption [7, 8]. More recently, a study integrating whole-genome sequencing and metabolomics determined that one third of PKU carriers had elevated Phe levels demonstrating a potentially pathogenic association with certain genetic variants [9]. A more detailed overview of preliminary research on health and metabolic effects among PKU carriers has recently been summarized by Khan et al. [10]. Treated PKU patients adherent to protein dietary restrictions often times still present with sympotoms such as difficulty concentrating, headaches, difficulty with short-term memory, impulsivity and others [11, 12]. Due to the metabolic pertubations that have been consistently reported in PKU carriers [6–8, 13], it is plausible to hypothesize that a clinical intermediate phenotype in carriers may present similarly. However, research has yet to thoroughly examine health outcomes of PKU carriers.

As an autosomal recessive condition, there have been over 1500 identified bi-allelic variants within the *PAH* gene that have been linked to PKU [14]. These variants are found at different loci and are associated with varying severity of the condition, mild hyperphenylanemia (HPA) being the least severe, followed by mild PKU and classical PKU being the most severe [14]. The most common pathogenic variants have been reported to occur in the following *PAH* locations: I65T, R261Q, G272*, R252W, R261*, R408W, IVS12 + 1 G > A, Y414C, IVS10-11 G > A [14]. Additionally, tetrahydrobiopterin (BH_4_) is an essential cofactor required for proper PAH enzyme function and unrelated genetic variants impacting this cofactor can lead to mild HPA, a less severe diagnosis compared to mild or classic PKU [15].

In many areas, universal screening for PKU is routinely conducted on neonates shortly after birth via a heel prick Guthrie test using dried blood spots (DBS) collected on a filter paper card [16]. Currently, newborn screening for PKU is largely performed by high throughput tandem-mass spectrometry, where a presumptive mild HPA or PKU diagnosis is determined by elevated Phe levels in DBS extracts exceeding 120 µmol/L depending on juridiction [17]. Importantly, early detection and prompt treatment of PKU has been reported to be cost-effective with improved health outcomes for affected children as compared to later symptomatic detection [18]. Once a newborn is confirmed to be diagnosed with PKU, they are instructed to follow a strict diet which is low in Phe-containing foods and beverages in order to lower the risk of severe developmental delay, neurological impairment, and psychiatric symptoms resulting from Phe build-up in the brain [16, 19, 20]. Families of infants affected with PKU generally limit breastfeeding and use special amino acid formulas that are low in Phe in an effort to avoid malnutrition and growth impairments [20]. Routine monitoring of PKU patients is needed to ensure blood Phe levels are within the current target therapeutic range (120–360 µmol/L) for affected children [20]; however, treatment outcomes for PKU patients can remain sub-optimal due to poor dietary adherence, as well as there being different practices in the clinical management of PKU [21].

In contrast, PKU carriers generally are unaware of their genetic status unless they have a child or parent with PKU. Currently, there is limited research evaluating the health and metabolic impacts of PKU carriers despite early evidence to suggest that they may present with an intermediate phenotype [10, 22]. Our research aims to bridge this knowledge gap. The purpose of this article is to first and primarily, detail the study protocol for an ongoing multi-omics clinical intervention feeding trial that aims to evaluate the putative metabolic, physiological and cognitive effects of an oral Phe challenge on carriers of PKU compared to non-carriers. In addition, this article further aims to present preliminary data from a completed pilot feasibility study which was conducted in order to test the feasibility of the proposed methods for the larger clinical trial. Modifications to the original study protocol after completing this pilot study will also be discussed. Carriers of PKU will simply be referred to as “carriers” and non-carriers of PKU will be referred to as “non-carriers” throughout. The pilot study (NCT05958784) and full cinical trial (Phe for Me? A Precision Nutrition Clinical Trial of Metabolic, Cardiovascular, and Neurocognitive Responses to Phenylalanine Among Carriers and Non-carriers of PKU) are registered with clinicaltrials.gov (NCT06119048).

## Aims and hypotheses of the Phe for Me Trial

### Primary outcome, aim and hypothesis

The primary objective is to determine whether there is a significantly different change in inhibitory control (e.g., response inhibition, primary outcome) from baseline to 2-hours post-Phe consumption in carriers compared to non-carriers. It is hypothesized that carriers experience a significantly greater decline in response inhibition (or inhibitory control), following Phe consumption in contrast to the non-carrier group, in which we expect to observe no significant differences in response inhibition.

### Secondary outcomes, aims and hypotheses

Secondary outcomes include baseline measurements of mental health, including chronic depression, chronic anxiety, chronic impulsivity, and acute mood. Additionally, this study aims to evaluate cognitive functioning (i.e., specifically working memory and executive functioning,) and concentration levels of Phe, Tyr, and other metabolites measured in a targeted and untargeted manner in three different specimens (DBS, saliva, urine) among carriers and non-carriers. It is hypothesized that baseline measures of mental health, mood and cognitive functioning will be poorer in carriers compared to non-carriers, while baseline levels of Phe, Tyr, Phe/Tyr ratio and their related metabolites will not differ between the two groups.

In addition to comparing baseline measures between the two groups, other secondary aims of this study include evaluating whether PKU carriers relative to non-carriers exhibit significantly different changes in cognitive functioning (i.e., working memory and executive functioning), mood, as well as levels of Phe, Tyr, and certain other related metabolites, following consumption of a Phe supplement. It is predicted that there will be significant impairment in specific cognitive domains, higher Phe, lower Tyr, higher Phe/Tyr ratio and changes to other metabolites observed in carriers compared to non-carriers. Additionally, non-carriers are predicted to have an improvement in acute mood following Phe supplementation compared to carriers who are predicted to experience worsened mood. This study will further explore whether or not there are changes in blood pressure (BP) and heart rate (HR) from baseline to 1 hour and 2 hours following Phe consumption. It is predicted that there will be no significant differences in BP and HR responses between groups both at baseline and after Phe ingestion.

Moreover, this study will explore whether any postprandial cognitive functioning, mood, or cardiovascular outcomes are correlated with levels of Phe, Tyr, and other metabolites, and/or genotype. It is predicted that there will be trends indicating possible correlations between post-prandial cognitive functioning and mood, and the levels of Phe, Tyr, Phe/Tyr ratio and/or certain other metabolites. It is also predicted that changes in cognitive functioning and mood will be associated with genetic variation whereby those with a variant for classical PKU (more severe) will have greater cognitive decline and worsened mood scores after Phe consumption than those with variants for mild PKU or mild HPA (less severe). However, these analyses will be exploratory as the trial may be underpowered to detect such differences.

Lastly, the study will evaluate whether salivary and urinary Phe levels can be used as non-invasive biofluids to DBS extracts that may allow differentiation of responses to Phe ingestion between carriers and non-carriers. It is predicted that the salivary and urinary Phe levels may offer a more convenient sampling method that is more amenable to repeat sampling when measuring Phe at baseline and 2-hours post-Phe consumption. Lastly, symptoms from the oral Phe challenge will also be monitored and evaluated, including within and between group analyses.

## Overview of methods for Phe for Me Trial

This is a single arm, clinical intervention trial with participant stratification based on PKU carrier status. The study is registered with clinicaltrials.gov (pilot study: NCT05958784; full clinical trial: NCT06119048). Participants are being recruited through local PKU clinics and PKU organizations, as well as through snowball sampling.

Inclusion and exclusion criteria are detailed in Table 1. Participants will be screened and excluded from participating if they do not meet these criteria.

**Table 1:** Inclusion and exclusion criteria for participants

Inclusion	Exclusion
18 years of age or older	Diagnosed with PKU
Carriers or non-carriers of PKU	Diagnosed with a neurological disorder (e.g. Alzheimer’s disease, Parkinson’s disease, dementia)
	Pregnant or breastfeeding
	Have a baseline blood pressure > 150/100 or < 90/65
	Currently taking monoamine oxidase inhibitor anti-depressants
	Diagnosed with melanoma
	Diagnosed with kidney and/or liver disease
	Not comfortable fasting the morning of the study
	Body Weight > 150 kg
	Allergic/intolerant to citrus and/or oranges
	History of fainting during blood draw
	Answer 1, 2, or 3 to question #9 of the PHQ-9*

*PHQ-9 Question 9: “Thoughts that you would be better off dead or of hurting yourself in some way” Answer options; 0-Not at all, 1-Several days, 2-More than half the days, 3-Nearly every day https://www.apa.org/depression-guideline/patient-health-questionnaire.pdf

**Fig. 1:**
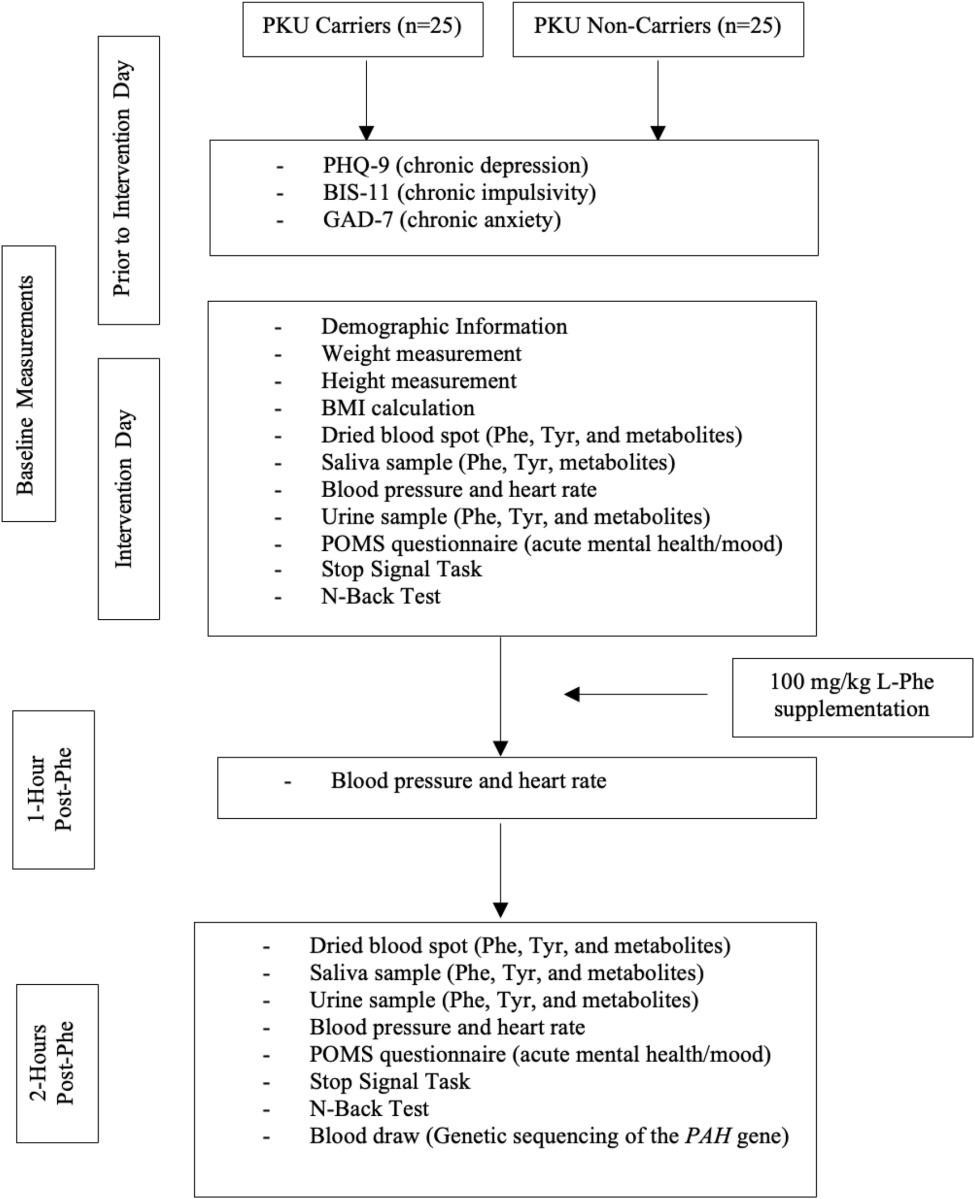
Study overview

Upon screening, eligible participants will undergo informed consent, before making a decision about participating in the study. An overview of the study protocol is presented in Fig. 1. Participants will be asked to complete validated chronic mental health assessment questionnaires using secure Qualtrics software prior to the intervention date. The mental health assessments will consist of the Patient Health Questionnaire (PHQ-9) [23], the General Anxiety Disorder (GAD-7) survey [24], and the Barratt Impulsiveness Scale (BIS-11) [25]. The PHQ-9 results will be reviewed prior to the intervention day and if participants answer 1, 2, or 3 to question 9 (see footnotes in Table 1) they will no longer be eligible to participate in the study and the researchers will contact the participant and their family physician given the duty to report (as detailed in the letter of information for participants).

The participants will be required to fast the morning of the study (i.e. no food or beverage consumption other than water) and arrive at The Human Nutraceutical Research Unit (HNRU) at the University of Guelph where the remaining data collection and L-Phe intervention will take place. Baseline measures will include height, weight, and body mass index (BMI) measurements and calculations as well as validated cognitive testing (N-Back Test and Stop Signal Task), the Profile of Mood State (POMS) questionnaire, a dried blood spot sample, a urine sample, a saliva sample, and a BP and HR measurement. Participants will also a demographic survey including information regarding age, sex, ethnicity, socioeconomic status, and will be asked to report any symptoms/side effects they may experience following L-Phe consumption. Upon completion of all baseline and follow-up measures, a blood draw will be performed by a trained phlebotomist, and shipped to Newborn Screening (NBS) Ontario for genetic sequencing. Further details on the methods used to collect this data are detailed below.

Phe supplementation (obtained from PureBulk®) dosed at 100 mg/kg body weight will be calculated, and measured, and given to each participant mixed in 125 mL water + 125 mL orange juice. Participants will remain sedentary in the HNRU until follow-up tests take place. BP and HR will be measured and recorded 1-hour and 2-hours postprandial. Two-hours postprandial, all cognitive tasks, the POMS questionnaire, dried blood spot, urine, and saliva samples will be repeated.

Participants will be provided low-Phe snacks and beverages once final measurements are taken and will be asked to remain in the research unit until they feel comfortable to leave. They will also be asked to avoid consuming protein and aspartame containing foods for the remainder of the day.

## Sample size

A sample size calculation was conducted to determine the sample size required to detect significant differences in change in stop signal reaction time (SSRT) (primary outcome) between the two groups (carriers vs. non-carriers). Using the pilot study data indicating a mean difference of 21.17 between groups for SSRT change scores, and pooled standard deviation of 29.18, with an alpha of 0.05 and power of 80%, a sample size of *n* = 18 participants per group (*n* = 36 participants total) is required to detect significant differences in the primary outcome. In addition to the sample size calculation, we further considered the possibility of participant dropouts, the feasibility of recruiting > 36 participants, and the ability to improve study power/the ability to detect significant differences in secondary and exploratory outcomes. Therefore, we aim to recruit a total sample of *n* = 50 (*n* = 25 per group) for this clinical trial.

## Chronic mental health questionnaires

Prior to the intervention day, participants will be asked to fill out the following chronic mental health questionnaires: BIS-11, PHQ-9 and GAD-7 [23–25]. These questionnaires will be completed using Qualtrics survey software. The BIS-11 is a validated questoinnaire used to assess impulsivity [25]. The PHQ-9 is a validated questionnaire used to assess depression [23] and the GAD-7 is a validated assessment of anxiety [24]. These questionnaires are used clinically as screening tools for depression, anxiety, and impulsivity.

## Blood draws and next generation sequencing of *PAH* Gene

For each participant, 2 × 5 mL of blood will be drawn for genetic analyses. Ethylene-diaminetetraacetic acid (EDTA) sample collection tubes will be used for blood draws and will be stored at room temperature until analyses. The samples will then be sent to Newborn Screening Ontario (NSO) for next generation sequencing (NGS) of the *PAH* gene to detect carrier status.

NGS will be conducted by custom capture, and sequenced on a NovaSeq6000 with analysis restricted to the *PAH* gene. Sanger sequencing will be used to supplement NGS for sequence targets with < 20X read depth for any coding base, and/or variant validation. Copy Number Variation (CNV) analysis will not be performed. The capture methodology used along with NGS does not detect large deletions, insertions, chromosomal abnormalities or large rearrangements; this is a limitation. This test was developed and its performance characteristics determined by NSO as required by Accreditation Canada and ISO 15189 PlusTM accreditation standards. This laboratory has established and verified the test’s accuracy, and this test is used for clinical purposes.

Variant classification will be performed following the 2015 American College of Medical Genetics and Genomics Standards and guidelines for the interpretation of sequence variants [26]. Only variants categorized as pathogenic, likely pathogenic, or variant of uncertain significance (VUS) are reported. The analysis and interpretation is based on current knowledge of the genes targeted. All clinical reports are reviewed, interpreted, and authorized by a certified genetic counsellor and an accredited molecular director.

## N-Back test

The N-Back Test is a computer-generated, validated test used to assess working memory [27]. Working memory is where temporary storage of limited information is held to be used to perform cognitive tasks [28]. Instructions will first be read out loud to the participant by a trained researcher, and the participant will have an opportunity to ask questions for clarification purposes. Following this, the participants will complete the test. Participants will be shown a series of images/stimuli and will then be asked to recall if the current stimuli they are looking at is the same as a stimulus that was shown “n” stimuli ago [27]. Higher scores (more correct answers) indicate better working memory [27].

## Stop signal task

The Stop Signal Task is a validated test for assessing response inhibition and reaction time [29]. This task will be used to measure SSRT, stop signal delay (SSD), and variability in reaction time (ICOV) [29]. The Stop Signal Task is generated and scored by a computer to assess reaction time and impulse control [29]. Instructions will first be read out loud to the participant by a member of the research team. Participants will have their fingers laying over the left and right arrow keys and once an arrow shows on the screen the participant must click the corresponding arrow as soon as possible [29]. However, an auditory cue: “beep”, occurs after the arrow appears on a subset of trials indicating that the participant should not press any keys (i.e. they should stop reacting) [29]. A quicker SSRT indicates better impulse control [29].

## Biological sample collection and metabolomic analyses

DBS filter paper cards, urine and saliva samples will be stored at −80 °C to ensure maximum stability of metabolites and prevent pre-analytical sources of bias [30]. These frozen samples will be transferred to the Britz-McKibbin laboratory at McMaster University to be analyzed for the quantification of Phe, Tyr, Phe/Tyr ratio and other metabolites associated with Phe ingestion and carrier status using a targeted and untargeted metabolomics data workflow. High-throughput metabolite profiling of urinary Phe, Tyr and other Phe-related catabolites from PKU patients was previously demonstrated when using multisegment injection-capillary electrophoresis-mass spectrometry (MSI-CE-MS), which demonstrated good mutual agreement to a validated commercial amino acid analyzers used in a clinical setting [31]. A large panel of amino acids, acylcarnitines and nucleosides can also be measured from a single DBS punch (3.2mm diameter) extracts using MSI-CE-MS as required for confirmatory diagnosis of presumptive screen-positive neonates with PKU and various other in-born errors of metabolism [32], including cystic fibrosis [33]. Briefly, DBS, urine and saliva samples will be diluted in deionized water containing matching stable-isotope internal standards and then ultrafiltrated using a 3kDa molecular weight cut-off filter to remove protein prior to MSI-CE-MS. Calibration curves will enable quantification of Phe, Tyr and Phe/Tyr ratio concentration levels from DBS extracts, saliva and urine (creatinine normalized) specimens, which will be further supported by analysis of standard reference samples (NIST) and pooled quality control (QC) samples. A standardized metabolomics protocol will be applied for targeted and untargeted metabolite profiling when using MSI-CE-MS under positive and negative ion mode detection with full-scan data acquisition as described elsewhere [34]. An accelerated data workflow for metabolite authentication and biomarker discovery will be applied after filtering spurious signals, background ions and redundant molecular features using a new software tool for automated data pre-processing of multiplexed separation in MSI-CE-MS [35].

Blood collection for metabolomics analyses will be done via DBS capillary finger prick sampling. Participants will be asked to wash their hands and then an alcohol pad will be used to disinfect the finger. The finger will then be pricked using a Unistik 3 single-use lancet by removing the cap and pressing it firmly against the disinfected fingertip. The first drop will be wiped away and with the following drops, a blood spot will be made on a Whatman 903 Protein Saver Card. Pressure and heat may be used to stimulate blood flow however, “milking” the finger will be avoided to prevent hemolysis. This will be repeated 2-hours postprandial. The blood spots will be left to dry for two hours at room temperature before closing the card and storing in an airtight Zip lock bag at −80 °C.

Random single-spot urine samples will be collected into a sterilized container by the participant. The collection will then be mixed well by inverting the container back and fourth ten times, and 1 mL or urine will be transferred into a 1.7 mL polypropylene vial and stored at −80 °C. These steps will be repeated 2-hours post-supplementation.

Unstimulated saliva samples will be collected using a passive drool approach, whereby participants will allow saliva to drip through a straw and into a 1.7 mL polypropylene vial with a minimum volume of 1.0 mL. This approach will be repeated two hours post-supplementation. The vials will be stored at −80 °C.

## Acute mental health (mood) questionnaire

The POMS questionnaire measures mood stability and is a standard validated psychological test [36]. Participants will be given a list of words/phrases and will be asked to respond based on their acute mood. Examples of words/phrases include, “confused”, “clear headed”, or “sorry for things done,” among others. Participants will then respond with either “not at all”, “a little”, “moderately”, “quite a bit”, or “extremely” [36]. Six different mood profiles will then be scored by the computer software. The different profiles, with the normative ranges for results indicated in brackets, include: “Anger” (3.6–9.91), “Confusion” (4.00–7.38), “Depression” (3.11–8.67), “Fatigue” (5.37–8.16), “Tension” (5.66–9.62), and “Vigour” (15.64–18.51) [36].

## Blood pressure and heart rate

BP and HR will be measured using the validated Omron 907XL Professional Intellisense BP Monitor [37]. An appropriate size cuff will be placed over the right arm brachial artery and three oscillometric measurements will be taken at each time point and the average of the three will be calculated and used for data analysis purposes. During each measurement, participants will be asked to remain seated, with their feet flat on the floor and uncrossed and to refrain from speaking. The arm will be supported at heart level.

## Statistical analyses

The preliminary statistical analysis plan will include pooled analyses of all carriers vs. all non-carriers, as well as stratified analyses for carriers of classical PKU variants vs. milder PKU variants (in the carriers’ group) as determined by two existing credible databases [38, 39] including comparisons with non-carriers. Sex-stratified analyses will also be conducted. SPSS version 30.0.0.0 [172] will be used to conduct the statistical analyses. The statistical analysis plan assumes that the data will be normally distributed, but revisions to the proposed plan (e.g. the use of non-parametric tests) will be made accordingly as needed. Descriptive statistics will be used to first summarize the data among each group. Analyses of covariance (ANCOVA) will be used to compare baseline demographic variables, mental health and cognitive outcomes as well as levels of Phe, Tyr and their ratios and related metabolites between the two groups (carriers and non-carriers). We will evaluate if there are any between-group, sex-linked or *PAH* variant-linked differences in the baseline measures (e.g. stratification of *PAH* variants based on those associated with classical PKU vs. mild PKU vs. hyperphenylalaninemia) as well as 1-hour and 2-hour post-Phe outcomes. Linear regression models and repeated measures-ANCOVAs will be used to examine relationships between changes in the above-mentioned dependent variables from baseline to 2-hours post-Phe consumption. Adjustment for possible confounders will be conducted as needed. Multivariate and univariate statistical analyses will be used to identify and rank order a panel of metabolic signatures that differentiate PKU carriers from non-carriers after multiple hypothesis correction and covariate adjustments, including their correlation with mood and cognitive test scores. Phe and Phe/Tyr concentrations will be compared to normal reference ranges in an age/sex-matched non-PKU population, and a Pearson correlation analysis will be performed to explore the association between Phe and Tyr concentrations in matching DBS extract, saliva, and urine samples from the cohort. Pearson correlation analysis will also be used to compare Phe and Tyr concentrations with clinical outcomes. These analyses will be run pre and post-Phe consumption.

## Pilot feasibility study for the Phe for Me trial

### Aims and methods

Approval for the pilot study was obtained from the University of Guelph Research Ethics Board (approval #23–03-017). Methods were largely comparable to those described above, however some changes to the protocol were made following completion of the pilot study and are further detailed below. For example, the pilot study did not include genetic testing to determine carrier status (which is being incorporated into the full clinical trial). Instead, participants had to be known carriers or non-carriers of PKU (e.g. individuals with a child diagnosed with PKU or who had previously undergone genetic testing for PKU carrier status). The purpose of the pilot study was to test the feasibility of the study methods prior to conducting the full trial on a larger sample, as well as to identify possible trends in the data in order to inform outcomes of the full trial (while highlighting that the pilot study is underpowered to detect statistically significant differences, and the main purpose of conducting the pilot study was simply to evaluate protocol feasibility).

Upon testing the feasibility of the methods in the pilot study (*n* = 7), minor revisions to the original methods were made in order to strengthen the methods of the full trial. First, the *L*-Phe supplement was mixed with 1 cup of water and 1 tsp of sugar for the pilot study however, due to the extreme bitter taste of this beverage, the supplement will be mixed with 125 mL water and 125 mL orange juice in the full trial. Exclusion criteria was also revised for the full clinical trial. Specifically, the following were added: orange and/or citrus allergy or intolerance, body weight > 150 kg, BP < 90/65, and history of fainting during blood draw [40]. Furthermore, HR was added as an additional outcome at baseline, 1-hour and 2-hour post-Phe consumption, and the BIS-brief was changed to the complete BIS-11 in the full clinical trial. Finally, rather than untargeted metabolomics analyses, a targeted approach will be used to provide a focused analyses of all desired metabolites from the biological samples in the full clinical trial. All other methods noted in the above-mentioned protocol for the full Phe for Me clinical trial were the same in the pilot study.

## Results

**Fig. 2:**
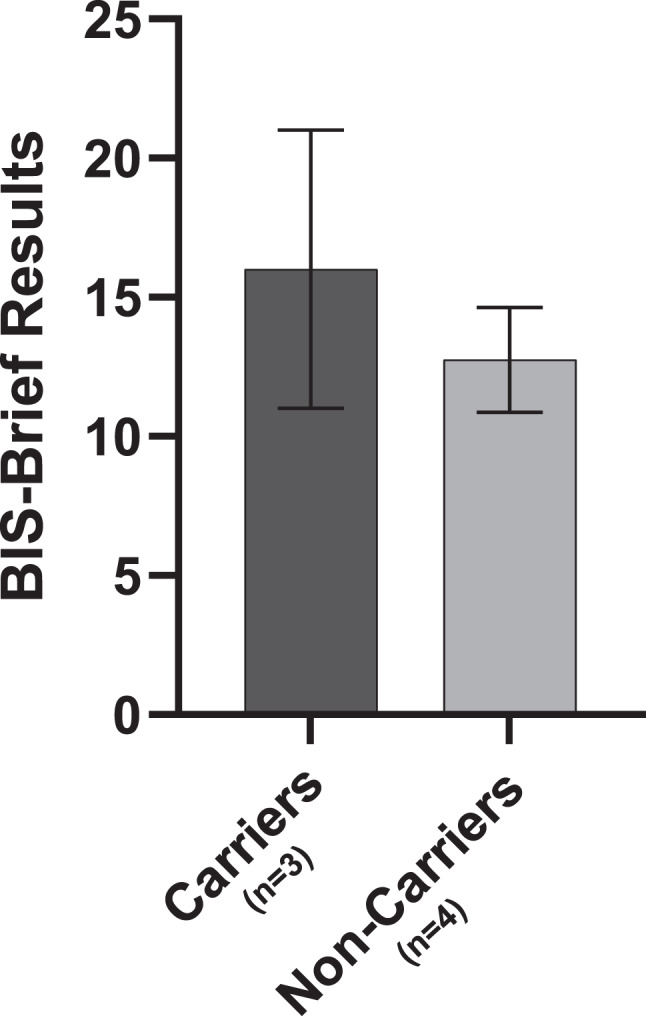
Chronic impulsivity scores (BIS-Brief results) in PKU carriers and non-carriers

**Fig. 3:**
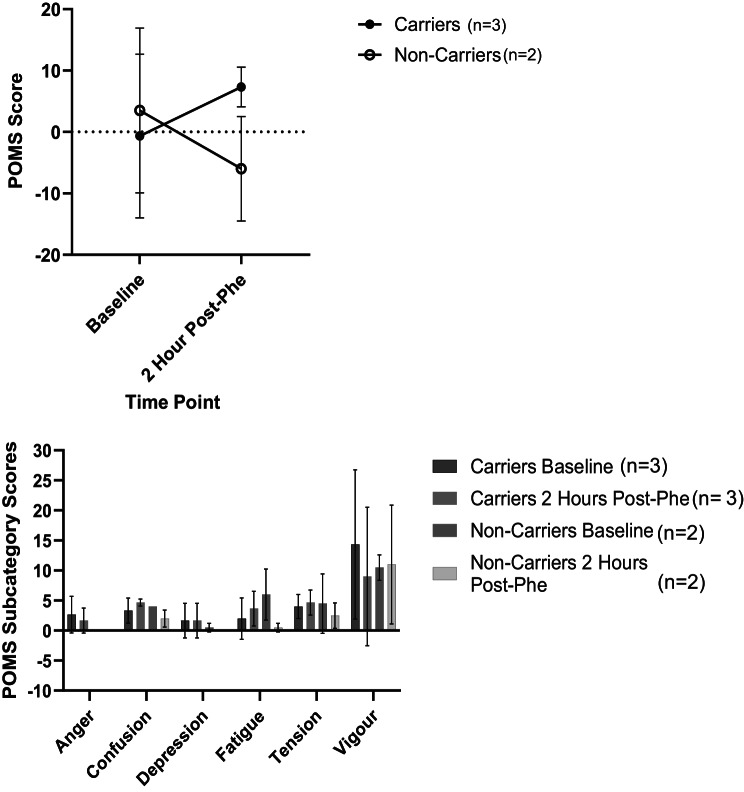
Acute mood scores (POMS results) pre and post Phe consumption among PKU carriers and non-carriers

**Fig. 4:**
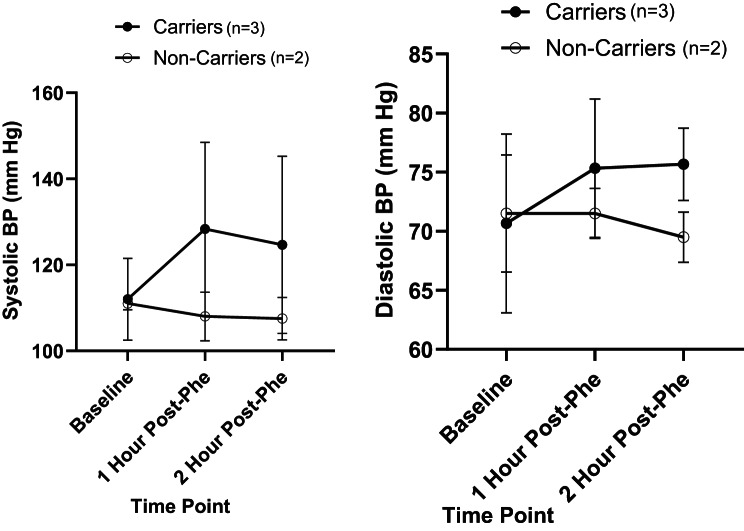
Systolic and diastolic BP pre and post Phe consumption among PKU carriers and non-carriers

The results of the pilot feasibility study are detailed below, noting that the primary purpose of this study was to test the feasibility of the methods rather than detect significant differences in the data. As such, statistical analyses were not performed and results of our primary and secondary outcomes are simply indicative of trends in the data which were observed, and could likely change in the full trial, in which we aim to recruit a larger sample size. These results should not be used to make inferences about the data or inform clinical practice including dietary recommendations or health/cognitive risks for carriers of PKU. Results of the cognitive tasks, acute mood questionnaire, urinary, salivary and DBS extract derived metabolites, chronic mental health status, blood pressure, participant demographic information and reported symptoms/side effects are detailed in Additional Tables 1, 2, 3, 4, 5, and 6 as well as Figs. 2, 3, and 4. A total of *n* = 7 participants (*n* = 3 carriers, *n* = 4 non-carriers) enrolled in the pilot study. 100% of participants were Caucasian and consisted of a combination of 42.9% males and 57.1% females, between the ages of 18 and 76. Two participants (both non-carriers) were lost to follow-up; the inclusion/exclusion criteria were revised as indicated above in order to minimize loss to follow-up in the larger Phe for Me Clinical Trial.

### Chronic mental health

PKU carriers tended to score higher (worse) on the BIS-Brief than non-carriers, which was the most notable difference observed between the carriers and non-carriers in terms of chronic mental health outcomes (Additional Table 4 and Fig. 2).

### Cognition and acute mood

PKU carriers exhibited an increase in POMS total mood scores (indicative of worsening mood) whereas non-carriers demonstrated a decrease (indicative of improving mood) 2 hours after Phe supplementation (Fig. 3 and Additional Table 1). Overall, there was an 8-point absolute change in POMS mood scores among carriers, whereas non-carriers exhibited a −9.5-point absolute change. Honing in on POMS sub-categories, non-carriers exhibited decreases (improvements) in levels of depression, fatigue, and tension scores following Phe intervention. Contrarily carriers demonstrated increases (worsening) of these same subcategories following the Phe intervention. PKU Carriers also scored notably lower (worsened) at the follow-up in the only positive emotion sub-category, vigour, compared to baseline whereas, non-carriers remained similar.

The results from the N-Back Test (to evaluate working memory) were also similar between carriers and non-carriers (Additional Table 1). As the N-Back difficulty increased (as it transitioned from 0-back to 1-back to 2-back) both groups’ scores worsened both at baseline and 2 hours post Phe supplementation. Overall, the change in scores tended to be comparable in carriers and non-carriers at all difficulty levels. The most notable difference was the change in 2-back targets (i.e., a measure of accuracy) in which there was a greater score reduction indicating worsening working memory in carriers than in non-carriers.

Lastly, the results of the Stop Signal Task demonstrated a greater decline in SSRT observed in carriers compared to non-carriers. While the ICOV score decreased slightly in carriers, it conversely increased slightly in non-carriers. Conversely, we observed a higher degree of stop signal delay in the non-carriers compared to the carriers.

### Blood pressure

Interestingly, trends in systolic and diastolic BP measures both demonstrated notable increases at 1-hour and 2-hour post L-Phe dose only in carriers. This contrasts with non-carriers, who tended to demonstrate either no change or a slight decrease in both systolic and diastolic BP 1-hour and 2-hours after the L-Phe intervention (Fig. 4). Overall, there was a 1.11 fold-change in systolic BP among carriers, whereas non-carriers exhibited a 0.97 fold-change. For diastolic BP, carriers demonstrated a 1.07 fold-change whereas non-carriers demonstrated a 0.97 fold-change.

### Metabolomic profiles

**Fig. 5:**
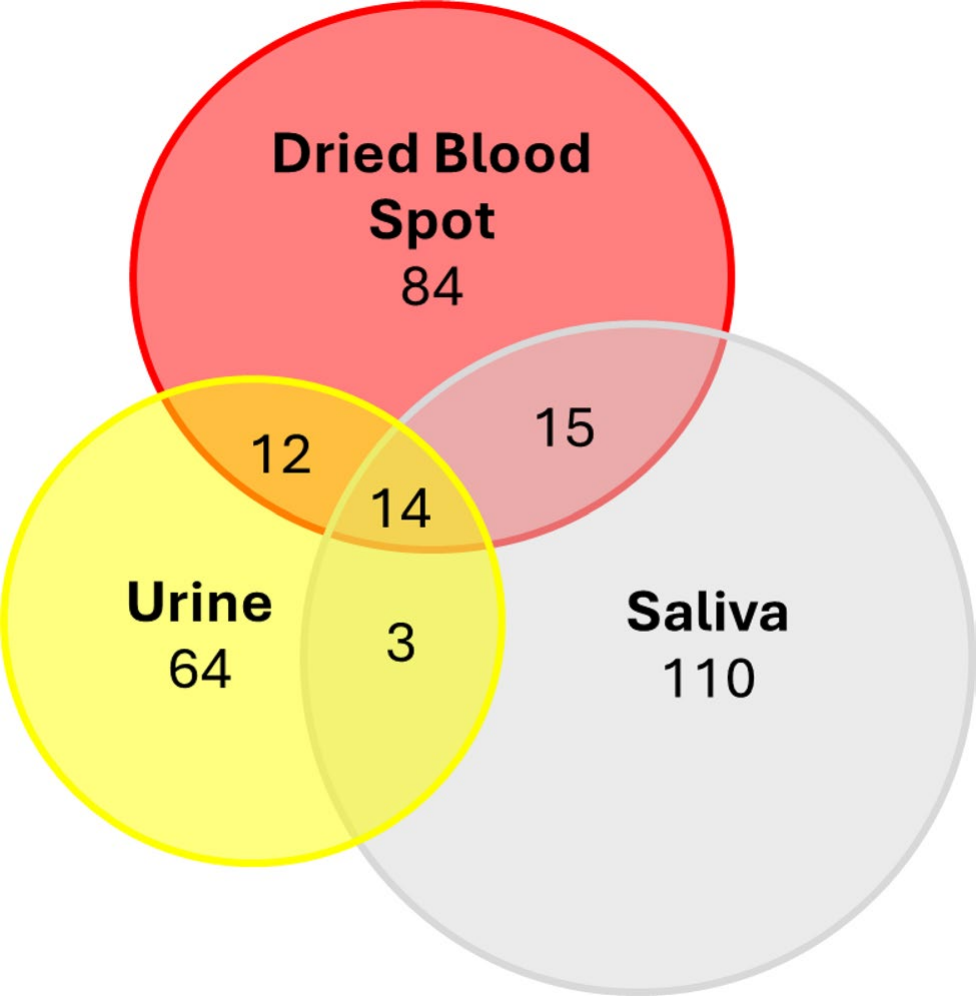
Venn diagram highlighting number of metabolites annotated by MSI-CE-MS in DBS extracts, urine and/or saliva

An untargeted metabolomics workflow using MSI-CE-MS characterized a total of 360 metabolite features (including unknown metabolites) which were consistently measured in all biospecimens collected from PKU carriers and controls, including DBS extracts, urine and saliva (Fig. 5). Fourteen metabolites were measured across all three specimens, including Phe or Tyr; others were unique to one specimen type. Overall, control charts for select recovery standards (e.g., F-Tyr) demonstrate good overall technical precision (CV < 8%).

**Fig. 6:**
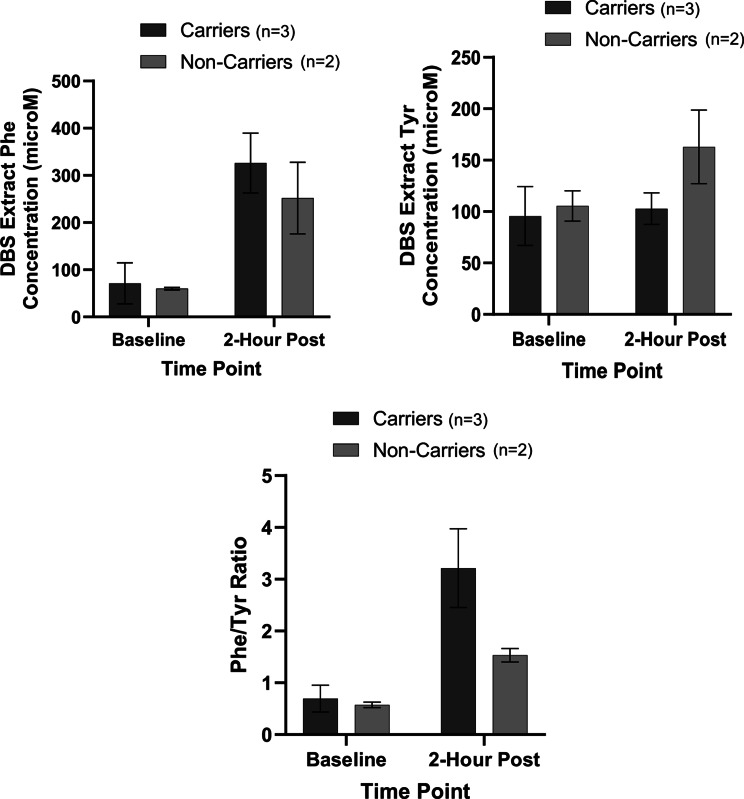
Phe, Tyr, and phe/Tyr ratio Pre and post Phe consumption among PKU carriers and non-carriers

A temporal analysis from baseline to 2-hour post Phe supplementation in this pilot study highlights that as expected we observed changes in Phe, Phe/Tyr as well as several unknown metabolites primarily in DBS extracts followed by urine (Additional Table 7, Fig. 6). Salivary derived metabolites did not appear to be responsive to the oral Phe challenge nor discriminate between carriers and non-carriers in this pilot study. Phe, Tyr, Phe/Tyr ratio and several other unknown metabolites showed a notable treatment response to Phe supplementation in both carriers and non-carriers (Additional Table 7, Fig. 6). There was a 4.63-fold change in Phe/Tyr ratio among carriers, but only a 2.67-fold change among non-carriers.

### Symptoms/side effects

Participants were ask to report any symptoms/side effects they may experience due to the oral Phe challenge test during the study visit. These reports included nausea and vomiting, tiredness/fatigue, sluggishnnes/heaviness, and mild headaches. Notably, these symptoms were only described by PKU carriers and the only reported symptom experienced by a non-carrier was fatigue.

## Discussion

Overall, we have demonstrated the feasibility of a clinical trial protocol through the successful pilot study completion. Minor changes to the protocol were implemented following pilot study completion in order to further optimize the methods to be used for the *Phe for Me* clinical trial with an anticipated goal sample size of *n* = 50. While trends in the data are highly preliminary at this time, it was interesting to observe some general data trends such as higher chronic impulsivity scores, as well as higher systolic and diastolic BP and worsening acute mood scores following the Phe intervention in carriers compared to non-carriers. We reiterate that the pilot study data is presented exclusively as descriptive statistics. Any trends observed at this stage are hypothesis-generating only and should not be used to make inferences about the data or inform any aspect of clinical practice. Pilot studies serve a distinct methodological purpose, and the use of formal hypothesis testing or inferential statistical analyses in this context is widely considered inappropriate [41–44]. The pilot study presented herein was conducted with the purpose of assessing feasibility, refining study design, and identifying potential logistical or methodological challenges, but not for evaluating treatment effects, which is consistent with current recommendations for pilot studies [41–44]. Reporting inferential statistics in an underpowered study leads to a high risk of type I and II errors and distorts the intended function of pilot work; as such, inferential statistics were not included in the present pilot study as these results would be unreliable and potentially harmful if used to guide decision-making [43, 45, 46]. As this pilot study was not hypothesis-driven and was underpowered, only descriptive statistics (means and standard deviations) were presented and inferential statistics were not conducted at this time to avoid the high risk of inappropriate conclusions being drawn from this nuanced work [41–44]. The larger *Phe for Me* clinical trial will provide us with further insights and inferential statistics on these preliminary findings.

If data trends observed in the pilot study are deemed to be statistically significant in the larger Phe for Me trial, these could be explained in the context of possible alterations in the PAH pathway. Dopamine and norepinephrine are both downstream products of Tyr metabolism and have been linked to positive mood [47]. In cases of a properly functioning PAH pathway, Phe supplements have been shown to improve acute mood related to an increase in the production of dopamine and norepinephrine [48]. In cases where the PAH pathway is impaired, the lack of production of these neurotransmitters would expectantly have a more negative effect on mood in carriers compared to non-carriers [2, 47]. Moreover, lower dopamine levels are also linked to higher rates of impulsivity patterns typically seen in addiction and attention deficit/hyperactivity disorder ADHD [49]. The decrease in dopamine would thus explain the trend towards higher chronic impulsivity in PKU carriers [49]. While pilot study data indicates potential differences in BP between carriers and non-carriers, the mechanism behind this is not clear. PKU patients have been reported to have higher BP than non-PKU individuals [50], representing the trend which we observed, whereby carriers experience higher BP following an oral Phe challenge compared to non-carriers.

Additionally, the cognitive tests showed small differences between carriers and non-carriers pre and post Phe loading. Since performance on these cognitive tasks can improve with practice [51], the assessment is based on differing patterns pre/post test between the carrier and non-carrier groups. The pilot study sample size was small, and the larger sample size used in the full clinical trial will allow us to more accurately evaluate these and all other outcomes.

Lastly, we observed higher Phe/Tyr ratios in carriers after Phe loading compared to non-carriers and responses to Phe loading appear to be best monitored by DBS specimen collection or alternatively urine as opposed to saliva. While possible distinctions between the two groups were found, a larger sample size will help us to better evaluate these outcomes. At a preliminary level it is intriguing to note that a Phe level > 130 μmol/L and a Phe/Tyr ratio > 3 is considered abnormal, on average, according to an international database of laboratories, as noted in the 2014 American College of Medical Genetics Practice Guidelines [52]. In the present pilot study, both carriers and non-carriers exceeded the 130 μmol/L limit post-Phe consumption (but had levels below 130 μmol/l when fasted). However, after a Phe challenge we observed in both carriers and non-carriers Phe concentrations within the American College of Medical Genetics and Genomics 2023 guidelines' recommended target range for blood Phe levels in adults with PKU ( < 360 μmol/L) [53]. Yet, the mean DBS Phe results after a Phe challenge was 74.03 μmol/L higher in the carriers group compared to the non-carriers. The carriers group also presented with a mean DBS Phe/Tyr ratio of 3.21 (thus exceeding normal limits) following Phe consumption, while non-carriers had a ratio of 1.53 (within normal limits). At baseline the carriers group had similar mean DBS Phe/Tyr ratios to the non-carriers (0.69 and 0.57 respectively; both within normal limits). Thus, the Phe/Tyr ratio tended to increase to a greater extent in carriers (4.63-fold change) compared to non-carriers (2.67-fold change) following Phe loading. A ratiometric measure improved discrimination of the treatment response to Phe loading in carriers from non-carriers since it reduces sources of between-subject variations from sampling, such as hematocrit in DBS specimens and hydration status in urine. It should be noted that levels of Phe/Tyr ratios exceeded normal limits in carriers only after Phe loading. While PKU diagnoses are not typically made following a high-Phe load, there is some clinical utility to Phe loading studies as they can be used to differentiate PKU from other causes of HPA unrelated to *PAH* variants, such as BH4 deficiency, through tetrahydrobpterin and Phe loading tests [55–56].

Moreover, investigating potential correlations between cognitive/cardiovascular results and metabolic findings is an objective of the full clinical trial. In the pilot feasibility study, correlational analyses were not conducted but it is interesting to note that the participants with the most notable improvement in mood scores were non-carriers and also had the lowest DBS Phe/Tyr ratios after Phe dosing. Contrarily the participants with the most notable increase in BP were carriers and expressed higher Phe/Tyr ratios compared to the non-carriers. Again, it is important to highlight that the sample size limits our ability to detect any statistically significant and/or clinically meaningful results; the trends observed in the data are simply “interesting” at this stage and the larger clinical trial is needed to corroborate these findings [47]. In addition, current work is underway to identify several unknown metabolites in urine and DBS extracts associated with differentiation of Phe loading responses in carriers than controls when using MS/MS with collisional-induced dissociation experiments [31, 34].

### Strengths and limitations

The *Phe for Me* clinical trial has many notable strengths including the investigation of both metabolic and clinical outcomes in PKU carriers compared to non-carriers, which has not been explored in a single study to date. Additionally, the trial compares carriers and non-carriers both at baseline and following a high Phe dose, thus, allowing the results to determine any chronic (specifically mental health) effects that carriers may experience as well as the acute consequences of high Phe doses. Moreover, comparing multiple types of biological samples can be beneficial to the PKU knowledge base and community, since aquiring blood samples from patients, especially young children, can be logistically challenging and discovering novel surrogate measurement methods of Phe could ease this process. There are some limitations, for example in recruitment as the most extreme cases of depression (those taking monoamine oxidase inhibitors and those who have thoughts of suicide or self-harm) must be excluded from participation for safety reasons. In addition, while the sample size will allow us to detect significant differences in the primary outcome, it may limit our ability to detect significant differences in secondary as well as exploratory outcomes. The important limitation from the pilot study is that it aimed to test the feasibility of the protocol and identify highly preliminary data trends but the sample size limited our ability to conduct statistical analyses, so results from the pilot data lack validity and reliability and should not be used to make inferences about the data or inform any aspect of clinical practice.

## Conclusion

Carriers of all autosomal recessive genetic conditions are currently considered “unaffected” despite literature indicating that they may actually be “moderately” affected, at minimum, metabolically [57]. PKU is a clinically variable condition, ranging from less severe HPA to more extreme classic PKU. Additionally, some PKU cases respond well to biopterin therapy while others do not [58]. This variability could extend to carriers, whereby those carrying more severe *PAH* variants may respond differently to an oral Phe challenge compared to those carrying less severe variants and compared to non-carriers. This clinical trial could demonstrate the connection between the physiological, psychological and metabolic effects that are hypothesized to be observed in PKU carriers. This trial may further provide ground-breaking insights into our understanding and treatment of carriers of autosomal recessive conditions, specifically PKU. For example, if the results demonstrate that carriers are intermediately affected it could provide insights into the need for more therapeutic treatments being directed towards this currently under-studied population, such as personalized lifestyle modifications (e.g., low protein/Phe diet). Conversely, it could provide a deeper understanding of carriers being unaffected, and not having specific health concerns. Overall, we need more research to better understand PKU carriers.

## Electronic supplementary material

Below is the link to the electronic supplementary material.

Supplementary Material 1

Supplementary Material 2

## Data Availability

Data available upon reasonable request.

## Abbreviations

PKU: Phenylketonuria
HPA: Hyperphenylalaninemia
PAH: Phenylalanine hydroxylase
Phe: Phenylalanine
Tyr: Tyrosine
DBS: Dried blood spot
BP: Blood pressure
HR: Heart rate
