# Supplementary material for: Study protocol and pilot study results for a clinical intervention trial of PKU carriers and non-carriers: the Phe for Me trial
Source: Orphanet J Rare Dis. 2026 Jan 19;21:18. doi: 10.1186/s13023-025-04131-2 (PMC12817628; doi:10.1186/s13023-025-04131-2)
Supplement: Supplementary file 1 — Supplementary Material 1 [file 13023_2025_4131_MOESM1_ESM.docx]

Additional Table 1. Cognitive and Acute Mood Outcomes Pre and Post Phe Consumption Among PKU Carriers and Non-Carriers

| Cognitive and Acute Mood Outcomes | | | | | | |
| --- | --- | --- | --- | --- | --- | --- |
|  | Carrier: Baseline (Scores, Mean ± SD) | Non-Carrier: Baseline (Scores, Mean ± SD) | Carrier: 2 Hour Post-Phe (Scores, Mean ± SD) | Non-Carrier: 2 Hour Post-Phe (Scores, Mean ± SD) | Carrier: Change from baseline to 2 hour post-Phe (Mean difference) | Non-Carrier: Change from baseline to 2 hour post-Phe (Mean difference) |
| N-Back Test | | | | | | |
| 0-Back Target | 1.00 _­­_± 0.00 | 1.00 ± 0.00 | 1.00 ± 0.00 | 1.00 ± 0.00 | - | - |
| 0-Back Distractors | 1.00 ± 0.00 | 1.00 ± 0.00 | 1.00 ± 0.00 | 1.00 ± 0.00 | - | - |
| 1-Back Target | 0.93 ± 0.06 | 1.00 ± 0.00 | 0.90 ­_­_± 0.10 | 0.97 ± 0.02 | -0.03 | -0.03 |
| 1-Back Distractors | 0.98 ± 0.03 | 0.99 ± 0.02 | 0.99 ± 0.01 | 0.92 ± 0.02 | 0.01 | -0.07 |
| 2-Back Target | 0.67 ± 0.32 | 0.75 ± 0.07 | 0.37 ± 0.31 | 0.63 ± 0.28 | -0.30 | -0.12 |
| 2-Back Distractors | 0.93 ± 0.07 | 0.99 ± 0.02 | 0.99 ± 0.01 | 0.95 ± 0.02 | 0.06 | -0.04 |
| Stop Signal Task | | | | | | |
| Stop Signal Reaction Time | 414.17 ± 108.35 | 270.00 ± 50.20 | 344.00 ± 132.86 | 221.00 ± 13.44 | -70.17 | -49.00 |
| Stop Signal Delay | 105.83 ± 118.57 | 192.50 ± 155.56 | 136.67 ± 221.73 | 300.00 ± 123.74 | 30.84 | 107.50 |
| Individual Coefficient of Variation | 0.19 ± 0.04 | 0.18 ± 0.01 | 0.18 ± 0.08 | 0.19 ± 0.01 | -0.01 | 0.01 |
| Profile of Mood State (POMS) Questionnaire | | | | | | |
| POMS Total | -0.67 ± 13.32 | 3.50 ± 13.44 | 7.33 ± 3.22 | -6.00 ± 8.49 | 8.00 | -9.5 |
| Anger | 2.67 ± 3.06 | 0.00 ± 0.00 | 1.67 ± 2.08 | 0.00 ± 0.00 | -1.00 | 0.00 |
| Confusion | 3.33 ± 2.08 | 3.00 ± 0.00 | 4.67 ± 0.58 | 3.00 ± 1.41 | 1.34 | 0.00 |
| Depression | 1.67 ± 2.89 | 0.33 ± 0.71 | 1.67 ± 2.89 | 0.00 ± 0.00 | 0.00 | -0.33 |
| Fatigue | 2.00 ± 3.46 | 5.33 ± 4.24 | 3.67 ± 2.89 | 1.33 ± 0.71 | 1.67 | -4.00 |
| Tension | 4.00 ± 2.00 | 3.67 ± 4.95 | 4.67 ± 2.08 | 2.67 ± 2.12 | 0.67 | -1.00 |
| Vigour | 14.33 ± 12.42 | 12.33 ± 2.12 | 9.00 ± 11.53 | 12.00 ± 9.90 | -5.33 | -0.33 |
| Sample size | *n=3* | *n=2* | *n=3* | *n=2* | *n=3* | *n=2* |
| Anger, confusion, depression, fatigue, tension, and vigour are all POMS subcategories. Lower POMS Total scores are indicative of a more positive mood. Lower scores in subcategories; anger, confusion, depression, fatigue, and tension, indicate a more positive mood. A higher score in vigour indicates a more positive mood. | | | | | | |

Additional Table 2. Metabolite Outcomes from Dried Blood Spot, Saliva, and Urine Samples, Measured in Concentrations (μmol/L) Pre and Post Phe Consumption Among PKU Carriers and Non-Carriers

| Metabolite Levels, Concentrations (μmol/L) – Dried Blood Spot | | | | | | |
| --- | --- | --- | --- | --- | --- | --- |
|  | Carrier: Baseline (Mean ± SD) | Non-Carrier: Baseline (Mean ± SD) | Carrier: 2 Hour Post-Phe (Mean ± SD) | Non-Carrier: 2 Hour Post-Phe (Mean ± SD) | Carrier: Change from baseline to 2 hour post-Phe (Mean difference) | Non-Carrier: Change from baseline to 2 hour post-Phe (Mean difference) |
| Phe Levels (μmol/L) | 71.23±43.45 | 60.15±2.76 | 325.93±63.38 | 251.9±75.66 | 254.7 | 191.75 |
| Tyr Levels (μmol/L) | 95.57±28.62 | 105.45±14.64 | 102.77±15.46 | 162.85±35.71 | 7.2 | 57.4 |
| Phe/Tyr | 0.69±0.26 | 0.57±0.05 | 3.21±0.76 | 1.53±0.13 | 2.52 | 0.96 |
| Sample size | n=3 | n=2 | n=3 | n=2 | n=3 | n=2 |
| Metabolite Levels, Concentrations (μmol/L) - Saliva | | | | | | |
|  | Carrier: Baseline (Mean ± SD) | Non-Carrier: Baseline (Mean ± SD) | Carrier: 2 Hour Post-Phe (Mean ± SD) | Non-Carrier: 2 Hour Post-Phe (Mean ± SD) | Carrier: Change from baseline to 2 hour post-Phe (Mean difference) | Non-Carrier: Change from baseline to 2 hour post-Phe (Mean difference) |
| Phe Levels (μmol/L) | 32.5±8.73 | 38.8±25.03 | 37.17±13.3 | 119.85±121.13 | 4.67 | 81.05 |
| Tyr Levels (μmol/L) | 79±5.1 | 64.4±24.75 | 75.3±12.4 | 69.15±9.12 | -3.7 | 4.75 |
| Phe/Tyr | 0.41±0.11 | 0.57±0.17 | 0.50±0.16 | 0.97±0.6 | 0.09 | 0.4 |
| Sample size | n=3 | n=2 | n=3 | n=2 | n=3 | n=2 |
| Metabolite Levels, Concentrations (μmol/L) - Urine | | | | | | |
|  | Carrier: Baseline (Mean ± SD) | Non-Carrier: Baseline (Mean ± SD) | Carrier: 2 Hour Post-Phe (Mean ± SD) | Non-Carrier: 2 Hour Post-Phe (Mean ± SD) | Carrier: Change from baseline to 2 hour post-Phe (Mean difference) | Non-Carrier: Change from baseline to 2 hour post-Phe (Mean difference) |
| Phe Levels (μmol/L) | 70.73±82.38 | 51.85±54.52 | 66.17±9.13 | 213.4±189.08 | -4.56 | 161.55 |
| Tyr Levels (μmol/L) | 85.8±73.87 | 135.2±112.43 | 35.33±7.90 | 117.7±57.13 | -50.47 | -17.5 |
| Phe/Tyr | 0.68±0.25 | 0.33±0.13 | 1.9±0.18 | 1.61±0.82 | 1.22 | 1.28 |
| Sample size | n=3 | n=2 | n=3 | n=2 | n=3 | n=2 |

Additional Table 3. Systolic and Diastolic Blood Pressure Outcomes Pre and Post Phe Consumption among PKU Carriers and Non-Carriers

| Blood Pressure | | | | | | | | | | |
| --- | --- | --- | --- | --- | --- | --- | --- | --- | --- | --- |
|  | Carrier: Baseline (Mean ± SD) | Non-Carrier: Baseline (Mean ± SD) | Carrier: 1 Hour Post-Phe (Mean ± SD) | Non-Carrier: 1 Hour Post-Phe (Mean ± SD) | Carrier: 2 Hours Post-Phe (Mean ± SD) | Non-Carrier: 2 Hours Post-Phe (Mean ± SD) | Carrier: Change from baseline to 1 hour post-Phe (Mean difference) | Non-Carrier: Change from baseline to 1 hour post-Phe (Mean difference) | Carrier: Change from baseline to 2 hour post-Phe (Mean difference) | Non-Carrier: Change from baseline to 2 hour post-Phe (Mean difference) |
| Systolic Blood Pressure (mm Hg) | 112.00 ± 9.54 | 111.00 ± 1.41 | 128.33 ± 20.13 | 108.00 ± 5.66 | 124.67 ± 20.60 | 107.50 ± 4.95 | 16.33 | -3.00 | 12.67 | -3.50 |
| Diastolic Blood Pressure (mm Hg) | 70.67 ± 7.57 | 71.50 ± 4.95 | 75.33 ± 5.86 | 71.50 ± 2.12 | 75.67 ± 3.06 | 69.50 ± 2.12 | 4.66 | 0.00 | 5.00 | -2.00 |
| *Sample size* | *n=3* | *n=2* | *n=3* | *n=2* | *n=3* | *n=2* | *n=3* | *n=2* | *n=3* | *n=2* |

Additional Table 4. Chronic Mental Health Outcomes Among PKU Carriers and Non-Carriers

| Chronic Mental Health | | | |
| --- | --- | --- | --- |
|  | Carrier (Scores, Mean ± SD) | Non-Carrier (Scores, Mean ± SD) | Difference Between Carriers vs. Non-Carriers (Mean difference) |
| PHQ-9 | 1.00 ± 1.00 | 1.50 ± 1.29 | 0.50 |
| GAD-7 | 1.00 ± 1.00 | 2.25 ± 2.23 | 1.25 |
| BIS-Brief | 16.00 ± 5.00 | 12.75 ± 1.89 | -3.25 |
| Sample size | *n=3* | *n=4** | *n=7** |
| PHQ-9 Interpretation, 0–4: None-minimal, 5–9 Mild, 10–14 Moderate, 15–19 Moderately severe, 20–27 Severe; GAD-7 Interpretation 0–4: minimal anxiety, 5–9: mild anxiety, 10–14: moderate anxiety, 15–21: severe anxiety; BIS-Brief: Higher score = more impulsive, lower score = less impulsive.  *n=2 of the non-carriers completed chronic health and demographic information but either did not continue participating (n=1) or their data had to be excluded from the interventional analyses due to protocol deviations (n=1). | | | |

Additional Table 5. Qualitative/Self Reported Outcomes of PKU Carriers and Non-Carriers

| **Qualitative Data: Side Effects Reported Following L-Phe Consumption** | | |
| --- | --- | --- |
|  | Carriers | Non-Carriers |
| Side effects | Nausea  Vomiting  Tiredness/fatigue  Sluggishness/heaviness  Head aches | Fatigue |
| Sample size | n=3 | n=2 |

Additional Table 6. Demographic Information of PKU Carriers and Non-Carriers

| Demographic Information | | | | | |
| --- | --- | --- | --- | --- | --- |
|  | Carrier (Mean ± SD) | Non-Carrier (Mean ± SD) | Non-carrier (Mean ± SD) | Difference between carriers vs. Non-Carriers (Mean difference) | Difference between carriers vs. Non-Carriers (Mean difference) |
| Sex | 33.33% Female  66.67% Male | 50% Female  50% Male | 75% Female  25% Male | - | - |
| Age | 54.33 ± 20.21 | 40.50 ± 7.78 | 33.00 ± 11.52 | -13.83 | -21.33 |
| Ethnicity | 100% Caucasian | 100% Caucasian | 100% Caucasian | - | - |
| Annual Income ($CDN) | 115,000 ± 58,949 | 145,000 ± 113,137 | 150,000 ± 65,701 | 30,000 | 35,000 |
| BMI (kg/m^2^) | 27.56 ±3.60 | 27.57 ± 0.55 | 23.44 ± 4.81 | 0.01 | -4.12 |
| Sample size | *n=3* | *n=2* | *n=4** | *n=5* | *n=7*^,^*** |
| Sex; 1 – Female, 2 – Male.  Ethnicity; Asian – 1, Black – 2, European/white – 3, Hispanic – 4, Indigenous – 5, Pacific Islander – 6, Multiple ethnicities/other – 7.  Note: for chronic mental health outcomes and demographic outcomes, two different sample sizes are reported. A sample size of n=5 encompasses participants who completed the entire study protocol. A sample size of n=5 encompasses participants who did not complete the full protocol but for whom chronic mental health and baseline data including demographic data was obtained.  *n=2 of the non-carriers completed chronic health and demographic information but either did not continue participating (n=1) or their data had to be excluded from the interventional analyses due to protocol deviations (n=1).  **Additional demographic analysis including 2 participants who’s data could not be included in the complete analysis | | | | | |
